# Toxic Dose prediction of Chemical Compounds to Biomarkers using an ANOVA based Gene Expression Analysis

**DOI:** 10.6026/97320630014369

**Published:** 2018-07-31

**Authors:** Mohammad Nazmol Hasan, Zobaer Akond, Md. Jahangir Alam, Anjuman Ara Begum, Moizur Rahman, Md. Nurul Haque Mollah

**Affiliations:** 1 Bioinformatics Lab, Department of Statistics, University of Rajshahi, Rajshahi-6205, Bangladesh; 2 Animal Husbandry and Veterinary Science, University of Rajshahi, Rajshahi-6205, Bangladesh; 3 Department of Statistics, Bangabandhu Sheikh Mujibur Rahman Agricultural University, Gazipur-1706, Bangladesh; 4 Agricultural Statistics and ICT Division, Bangladesh Agricultural Research Institute (BARI), Gazipur-1701, Bangladesh

**Keywords:** Dose, chemical Compounds, toxicogenomic biomarker, gene expression, One-way ANOVA, Tukey's HSD test

## Abstract

The aim of toxicogenomic studies is to optimize the toxic dose levels of chemical compounds (CCs) and their regulated biomarker genes. This is also crucial in drug discovery and development. There are popular online computational tools such as ToxDB and Toxygates to identify toxicogenomic biomarkers using t-test. However, they are not suitable for the identification of biomarker gene regulatory dose of corresponding CCs. Hence, we describe a one-way ANOVA model together with Tukey's HSD test for the identification of toxicogenomic biomarker genes and their influencing CC dose with improved efficiency. Glutathione metabolism pathway data analysis shows high and middle dose for acetaminophen, and nitrofurazone as well as high dose for methapyrilene as significant toxic CC dose. The corresponding regulated top seven toxicogenomic biomarker genes found in this analysis is Gstp1, Gsr, Mgst2, Gclm, G6pd, Gsta5 and Gclc.

## Background

The toxicogenomics is defined as the study of the relationship between the structure and activity of the genome (the cellular complement of genes) and the adverse biological effects of exogenous agents [1]. In the early stage of drug development the pharmaceutical companies are interested in evaluating the toxic or carcinogenic properties of new drugs including dose toxicity [2]. By gene expression fingerprints in response to different dose levels of drugs, we can explain about the underlying mechanism of toxicity that would be crucial to improve the drug development process [3]. Since, gene expression patterns changes results to its physiological condition changes [4, 5]. On the other hand, the main objective of toxicogenomics is to identify more reliable molecular predictors or toxicogenomic biomarkers of toxicity from the extensive amount of toxicogenomic data. The toxicogenomic biomarkers are a set of genes that are regulated by the influence of different dose levels of CCs or drugs. Identification of these biomarkers that are predictive for toxicity or to classify dose of CCs from the large-scale toxicogenomic data often suffers from robustness [6, 7]. Furthermore, proper identification of toxicogenomic biomarkers and their influencing dose of CCs/drugs is often depends on the selection of appropriate analytical tools. Most of the computational tools of toxicogenomic data are designed to identify the toxicogenomic biomarkers. But the characteristic of toxicogenomic data is that there are subsets biomarker genes which expression patterns are correlated over their regulatory dose of CCs [8]. Thus, simultaneous identification of biomarker genes and their influencing or toxic dose of chemical compounds are important in toxicogenomic study.

There are some online computational tools for the identification of toxicogenomic biomarker genes named ToxDB [9] and Toxygates [10]. Among these ToxDB a pathway based significant toxicogenomic biomarker gene identification tool for the selected chemical compound. But this tool cannot identify the significant dose level of CCs that regulate the expression pattern of biomarker genes. The other online computational tool toxygates can also identify the toxico-genomic biomarker genes using t-test and Mann-Whitney U test. Though this tool can rank the chemical compounds on the basis of the selected biomarker genes it has no statistical or probabilistic basis. Hence, we describe oneway ANOVA together with tukey's HSD test (post-hoc test) [11] for the identification of toxico-genomic biomarker genes and their regulatory or toxic dose of CCs respectively.

## Methodology

### Toxicogenomic Biomarkers and Toxic Dose of CCs

**Figure 1:**
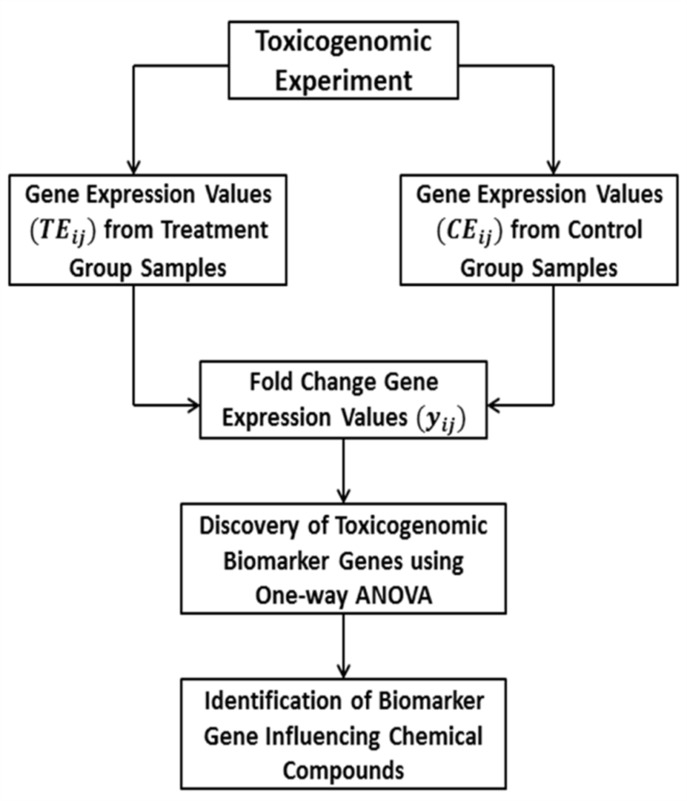
Flow chart for the identification of toxicogenomic biomarker genes and prediction of toxic doses of CCs.

Toxicogenomic studies profile transcriptional abundance to examine involving multiple dose levels and time points. Usually, toxicogenomic microarray experiment are designed in such an experimental setup in which gene expression is measured at its underlying factors, such as doses, time points or combination thereof from the treatment samples. There are also control samples concurrently to the treatment samples. The fold change gene expression yij data can be computed from the gene expression of the treatment group samples and control group samples. The step-by-step computational process for the identification of toxicogenomic biomarkers and their significant regulatory CCs are given in Figure 1. The fold change gene expression data can be obtained from the gene expression data of the treatment group and control group samples using the following formula:

*y^ij^ = TE^ij^ / CE^ij^* ---- (1)

Where, yij is the fold change gene expression value of the jth sample (replication) under the ith chemical compound-dose combination or treatment, TEij and CEij are the gene expression value of the treatment and control samples respectively of the jth sample under the ith treatment. One-way ANOVA is applied on the fold change gene expression data for the identification of toxicogenomic biomarker genes. Thereafter, Tukey's HSD (posthoc) test is applied for the identification of the toxicogenomic biomarker gene regulatory dose of chemical compounds or treatment.

### Toxicogenomic Biomarker Genes

Before identification of toxic dose of CCs it is necessary to identify toxicogenomic biomarker genes. For this purpose we have used one-way ANOVA model. Let us consider yijis the observed fold change gene expression value of a gene of the jth (i = 1, 2, ..., r) sample (replication) under the ith treatment (chemical compound-dose combination). The one-way ANOVA model for the fold change gene expression value yij can be expressed as follows:

y^ij^ = μ + α^i^ + ε^ij^ ---- (2)

Where, μ is the grand mean, αi is the ith treatment effect and εij is the random error term εij ~ N(0, σ2). The main objective of the above ANOVA model is to test whether all the main effects αi of the treatments are significantly different. Under null hypothesis, for testing the mentioned statement the statistic is used. On the basis of this statistic, if the null hypothesis becomes rejected (i.e., the treatment effects are significantly different) for a gene we declare that gene as toxicogenomic biomarker.

### Biomarker Gene Regulatory Dose of CCs

When the results of ANOVA indicate that the true treatment means are likely not all equal. The researcher interested to know which treatments are responsible for this difference. This can be performed comparing the treatment groups using the post-hoc test. There is no theoretical problem arises when comparing only two treatment groups. But when the test is performed for comparing many pairs of treatment groups at the same time, it will inflate the type-I error rate or family-wise error rate. We can define the family-wise error rate as the probability that at least one error is made on a set of tests or p (at least one error is made). The family-wise error is meant to capture the overall situation in terms of measuring the likelihood of making a mistake if we consider many tests, each with some chance of making their own mistake, and focus on how often we make at least one error when we do many tests. Nevertheless, there are many different statistical methods to perform the pair-wise comparisons; among those only the Tukey's Honestly Significant Difference (Tukey's HSD) method controls the family-wise error rate at your specified level (say 0.05 or 0.01) across many numbers of pair-wise comparisons. Thus, in this study we have chosen Tukey's HSD method for comparing treatments (compound-dose combinations) means to identify toxic or toxicogenomic biomarker regulatory dose of CCs.

## Results and Discussion

**Figure 2:**
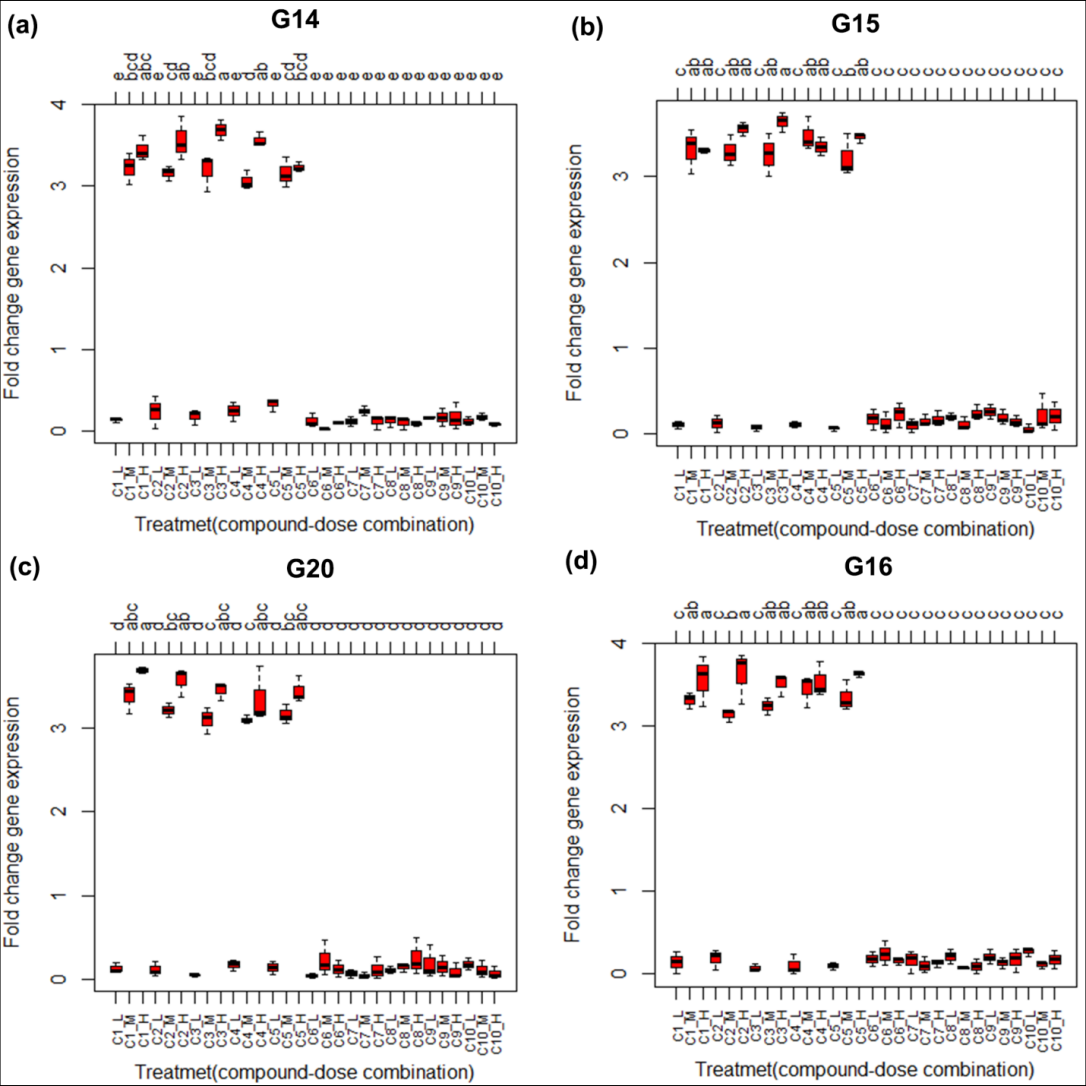
Boxplot for the treatments (chemical compound and dose combinations) along with lettering produced by Tukeys' HSD test for the top four significant biomarker genes in the simulated data.

**Figure 3:**
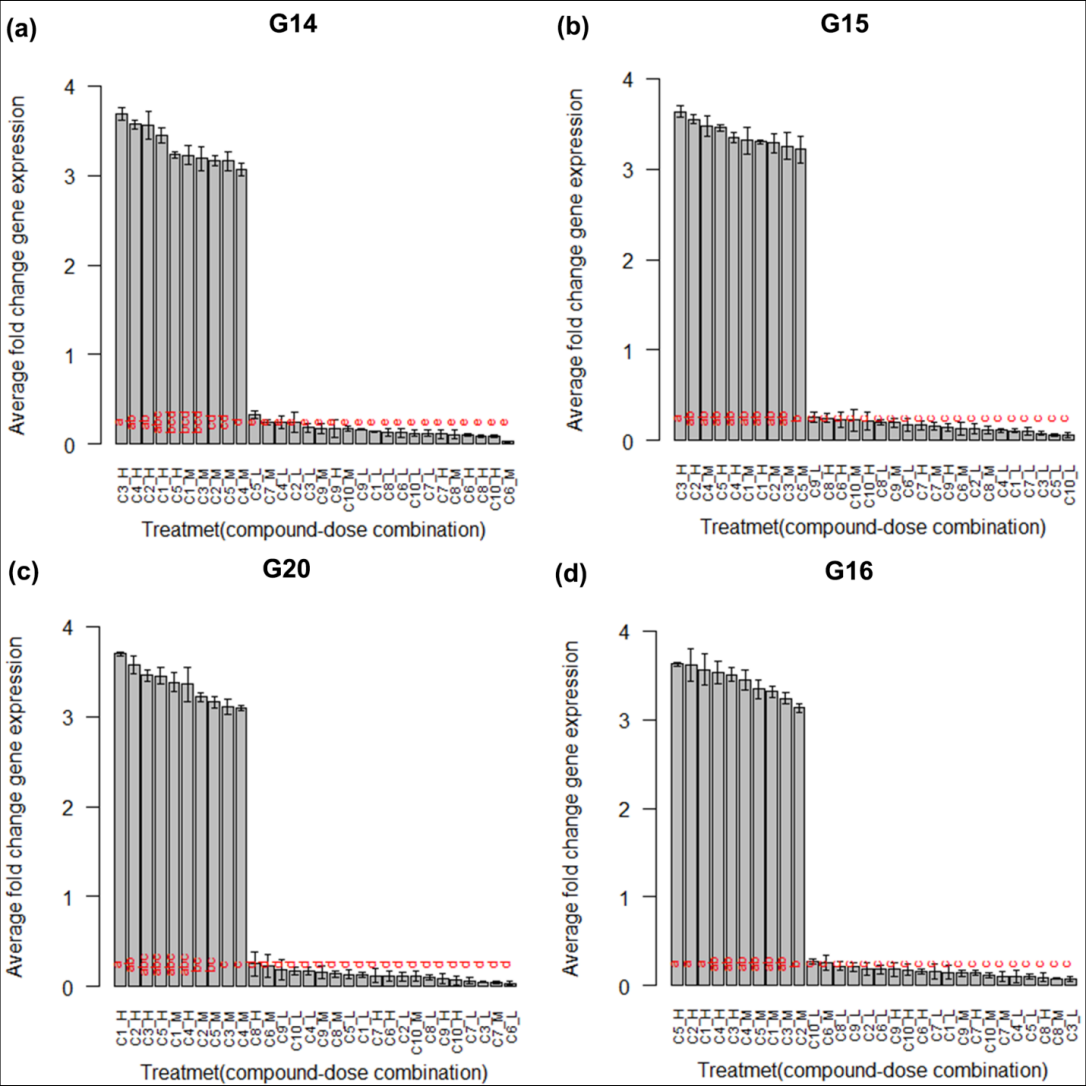
Barplot for the treatment (chemical compound and dose combination) means along with lettering produced by Tukeys' HSD test for the top four significant biomarker genes in the simulated data.

The important characteristics of toxicogenomic data are that the expression pattern of a subset of genes is correlated across a subset of chemical compounds (Afshari et al., 2011). Accordingly, in the pathway level gene expression data we assume that a subset of treatments alters the expression pattern of a particular subset of biomarker genes and the other treatments have no influence over the other subset of genes. In the typical toxicogenomic experiment there are treatment group and control group animal samples and gene expression data from both of the samples are collected. Later on, fold change gene expression can be computed using the equation (1). Therefore, we have simulated fold change gene expression data consisting of 50 genes and 30 treatments or compound-dose combinations using one-way ANOVA model (equation 2) to evaluate the aptness of the model in toxicogenomic data analysis. In the simulated data we have considered the genes (G1, G2, ..., G20) as toxicogenomic biomarker genes and high and meddle dose of the compounds (C1, C2, ..., C5) as the toxic dose or biomarker-influencing dose of CCs. For imitating the real life pathway level fold change gene expression data we have ordered the biomarker genes with respect to fold change expression value. The numerical order of the biomarker genes according to which the fold change gene expression data are generated is (G14, G15, G20, G16, G4, G17, G10, G8, G3, G18, G5, G7, G12, G13, G9, G19, G6, G1, G11 and G2). In this way we have simulated the fold change gene expression data 100 times and take their average to use in the final analysis. We have analyzed the simulated data using the one-way ANOVA and the genes for which the treatment effects αi are significantly different are considered as the toxicogenomic biomarker genes. The results of the ranked (based on p-value) significant biomarker genes are (G14, G15, G20, G16, G4, G17, G10, G8, G3, G18, G5, G7, G12, G13, G9, G19, G6, G1, G11, G2 and G29). It is observed that all the significant toxicogenomic biomarker genes are correctly identified according to their numerical order except the gene G29 as per the data is simulated. The boxplot and barplot along with the lettering obtained from Tukey's HSD test of the treatments are given in Figure 2 and Figure 3 respectively for the top four significant biomarker genes (G14, G15, G20 and G16). From these figures it is observed that the treatments (C1_low-C10_low, C6_medium-C10_medium, and C6_high-C10_high) which have no significant influence on the expression of the mentioned genes possess the same letter and the treatments (C1_high-C5_high and C1_medium-C5_medium) which have significant influence on the expression of the these genes possess the different letters. From these results it is observed that our proposed methods are efficient to discover the significant toxicogenomic biomarker genes and their regulatory treatments or toxic dose of CCs.

**Figure 4:**
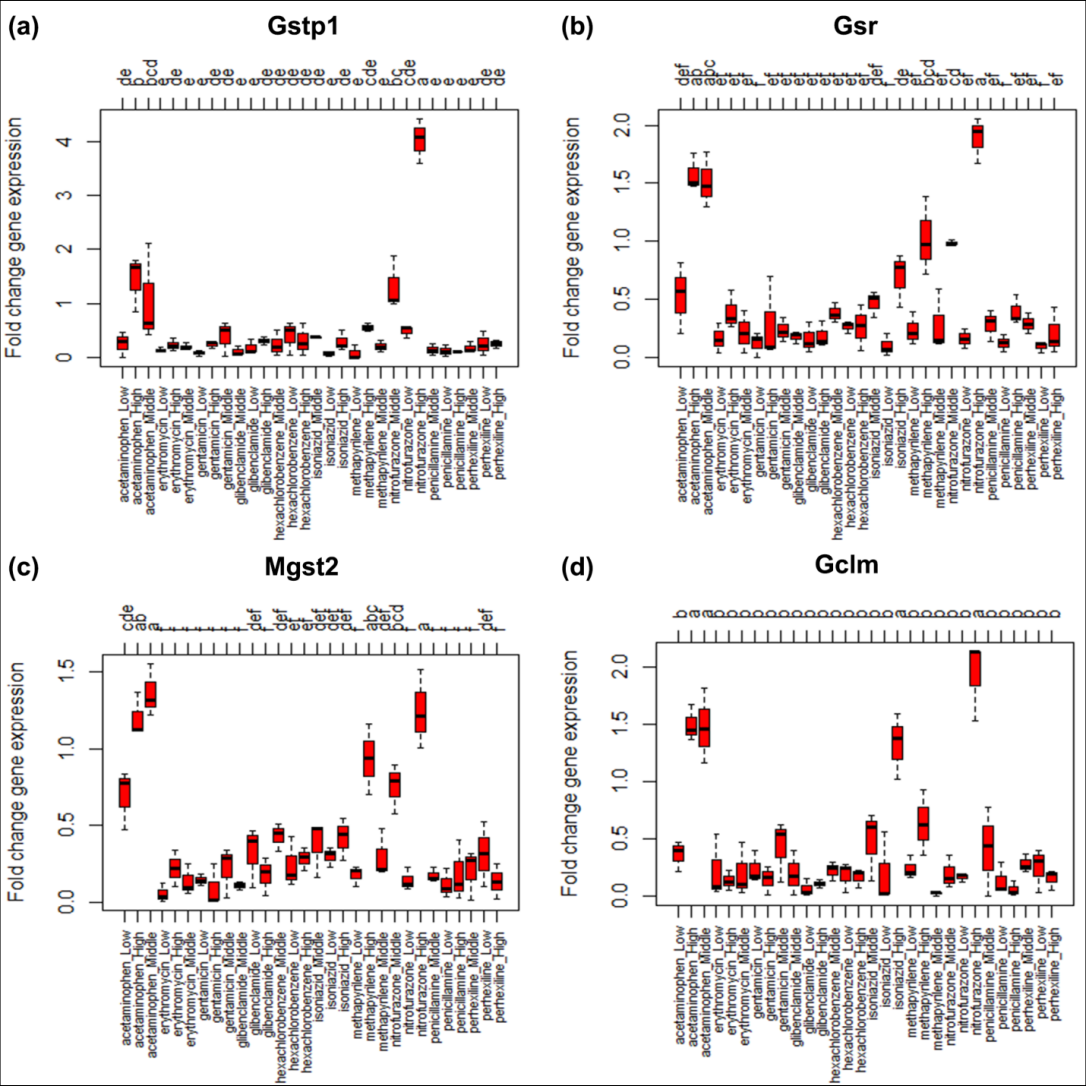
Boxplot for the treatments (chemical compound and dose combinations) along with lettering produced by Tukeys' HSD test for the top four significant biomarker genes in the glutathione metabolism pathway data.

**Figure 5:**
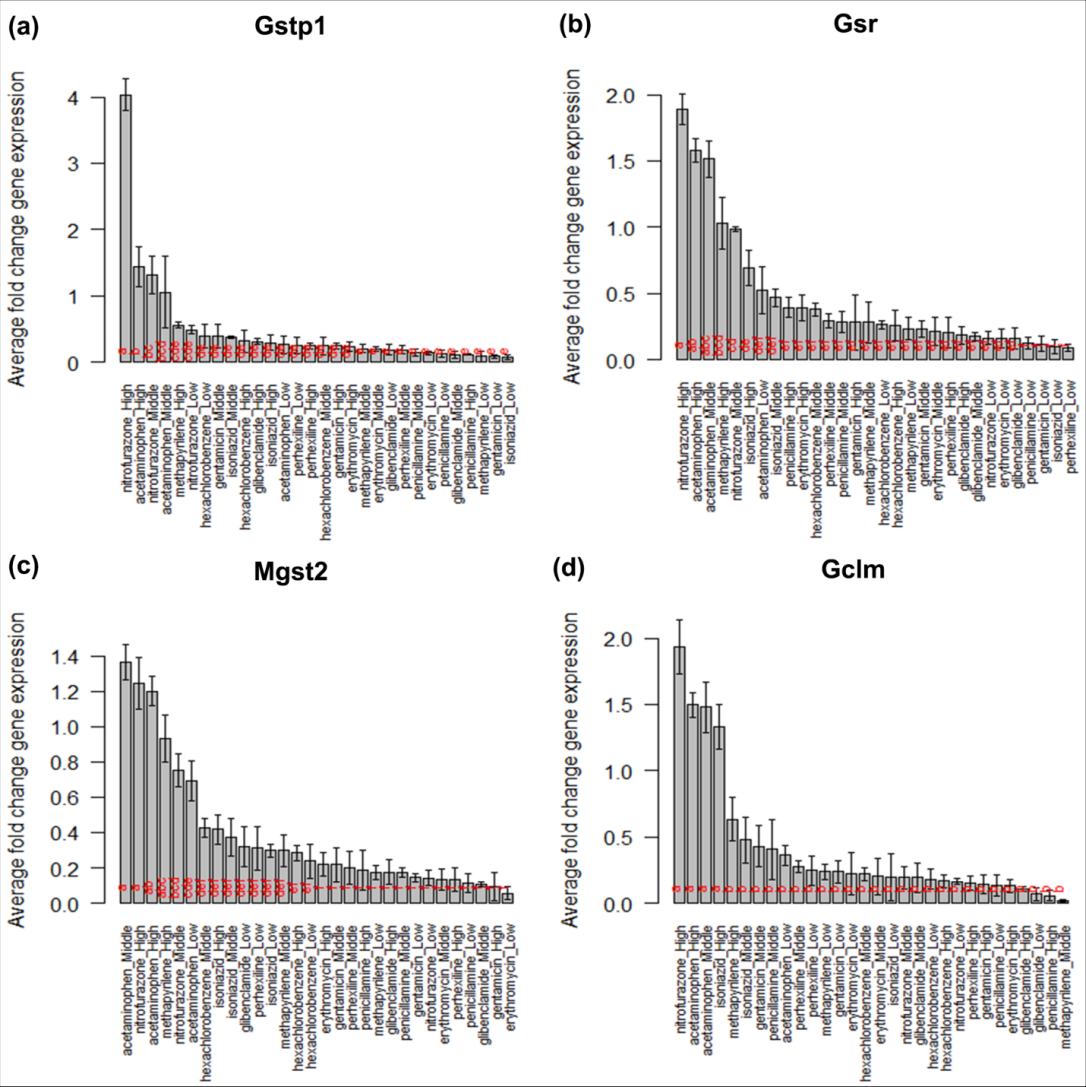
Barplot for the treatment (chemical compound and dose combination) means along with lettering produced by Tukeys' HSD test for the top four significant biomarker genes in the glutathione metabolism pathway data.

According to Nystrom-Persson et al. (2013) acetaminophen, methapyrilene and nitrofurazone are the glutathione (a major metabolite in detoxification process) depleting (toxic) compounds and non-glutathione depleting (non-toxic) compounds are erythromycin, gentamicin, glibenclamide, hexachlorobenzene, isoniazid and penicillamine. The toxicological effects of each compound are visible more clearly at 24-hour time point compared with 3 hour, 6 hour and 9 hour. Although Toxygates [10] provides six different datasets, in this article we have considered only rat/in vivo/liver/single data. For the discovery of pathway level toxicogenomic biomarkers and toxic dose of chemical compounds (treatments) using the described methods we have downloaded and analyzed the fold change gene expression data of glutathione metabolism pathway from toxygates (http://toxygates.nibiohn.go.jp/toxygates/#columns) for the mentioned glutathione affecting and non-glutathione affecting compounds along with dose levels (low, medium and high). The one-way ANOVA identified significant ranked (according to p-value) toxicogenomic biomarker genes are (Gstp1, Gsr, Mgst2, Gclm, G6pd, Gsta5, Gclc, Gstm4, Gss, LOC100912604/Srm, LOC100360180, Odc1, LOC100359539/Rrm2, Gstm1, Gsta2/Gsta5, Gsto1, Oplah, Idh1, Anpep, Gstm3, Rrm1, Gstm7, Gsta4, Mgst3, Gstt1, Apitd1/Cort/Kif1b/LOC100360180, Mgst1, Nat8, Gpx1, RGD1562107, Gstm2, Gpx2, Gpx4, Sms, Hpgds). The identified biomarker genes are functionally annotated using the online database DAVID [12] and the annotation results are given in Table 1. From the table it is observed that out of 35 identified toxicogenomic biomarker genes 30 are found statistically significant in the glutathione metabolism pathway. Among these biomarker genes Gstp1, Gsr, Mgst2 and Gclm are top four significant biomarker genes. The boxplot and barplot together with lettering produced by Tukey's HSD test for these biomarker genes are depicted in the Figure 4 and Figure 5. In the case of the biomarker gene Gstp1 the figures Figure 4A and Figure 5A show that nitrofurazone-high (a) is the most significant toxic treatment for regulating the gene Gstp1 and then acetaminophen high (b), nitrofurazone_middle (b) and acetaminophen_middle (bc). Similarly, for the biomarker gene Gsr Figure 4B and Figure 5B represent that nitrofurazone_high (a), acetaminophen_high (ab), acetaminophen_middle (ab), methapyrilene_high (bc) and nitrofurazone_middle (bcd) are the important significant toxic treatments that affect the expression pattern of Gsr. From the Figure 4C & 4D, Figure 5C & 5D it is observed that acetaminophen_ middle (a), nitrofurazone_high (ab), acetaminophen_ high (ab), methapyrilene_high (abc) and nitrofurazone_middle (abcd) affect the expression of the biomarker gene Mgst2 significantly and nitrofurazone_high (a), aceta-minophen_high (a), aceta-minophen_middle (a) and isoniazid_high (a) affect the expression of of biomarker gene Gclm. Here it should be mentioned that the letters within parenthesis after the treatments represents their significance for altering the expression of the respective gene. The results obtained from the proposed methods are also consistent from the other findings.

## Conclusion

Available online toxicogenomic data analysis tools ToxDB and Toxygates are suitable for the identification of biomarker genes alone. Hence, we describe a model for the identification toxicogenomic biomarker genes and their influencing treatments. Glutathione metabolism pathway data analysis shows high and middle dose for acetaminophen and nitrofurazone, as well as high dose for methapyrilene as significant toxic CC dose. The corresponding regulated top seven toxicogenomic biomarker genes found in this analysis is Gstp1, Gsr, Mgst2, Gclm, G6pd, Gsta5 and Gclc.

## Conflict of Interest

The authors declare no conflict of interests.

## Figures and Tables

**Table 1:** Functional annotation of KEGG pathway on the biomarker genes identified by one-way ANOVA for glutathione metabolism pathway data.

Term	Count	%	p-value	Genes
rno00480: Glutathione metabolism	30	88.24	1.21E-64	Anpep, G6pd, Gclm, Gstm7, RGD1562107, Sms, Gstm4, Gstm1, Apitd1/Cort/Kif1b/LOC100360180, Gpx2, Gstt1, Odc1, Gsta5, Gclc, Gpx4, Gsta2/Gsta5, Gpx1, Gsta4, LOC100360180, Rrm1, Idh1, Gstm2, Mgst3, Gsr, Gss, Gstp1, Mgst1, Mgst2, Oplah, Gsto1, Gstm3
rno00980: Metabolism of xenobiotics by cytochrome P450	15	44.12	7.42E-22	Gstm2, Mgst3, Gstm7, RGD1562107, Gstp1, Gstm4, Gstm1, Mgst1, Mgst2, Gsto1, Gstt1, Gsta5, Gsta2/Gsta5, Gstm3, Gsta4
rno00982: Drug metabolism - cytochrome P450	15	44.12	9.22E-22	Gstm2, Mgst3, Gstm7, RGD1562107, Gstp1, Gstm4, Gstm1, Mgst1, Mgst2
			7.67E-16	Gsto1, Gstt1, Gsta5, Gsta2/Gsta5, Gstm3, Gsta4
rno05204: Chemical carcinogenesis	15	44.12	3.92E-20	Gstm2, Mgst3, Gstm7, RGD1562107, Gstp1, Gstm4, Gstm1, Mgst1, Mgst2, Gsto1, Gstt1, Gsta5, Gsta2/Gsta5, Gstm3, Gsta4
rno01100: Metabolic pathways	11	32.35	0.0191	LOC100360180, Apitd1/Cort/Kif1b/LOC100360180, Anpep, Rrm1, Idh1, G6pd, Gclm, Odc1, Gclc, Sms, Gss, Hpgds
rno04918: Thyroid hormone synthesis	3	8.82	0.0279	Gpx2, Gsr, Gpx1
rno00590: Arachidonic acid metabolism	3	8.82	0.0385	Gpx2, Gpx1, Hpgds
rno01130: Biosynthesis of antibiotics	4	11.76	0.0511	LOC100360180, Apitd1/Cort/Kif1b/LOC100360180, Idh1, G6pd
rno01200: Carbon metabolism	3	8.82	0.0786	LOC100360180, Apitd1/Cort/Kif1b/LOC100360180, Idh1
